# 

**Published:** 1921-01-08

**Authors:** 


					Matrons' and Sisters'
Appointments.
Essex. County Hospital, Colchester.?Miss Winifred
Rowo has been appointed out-patient and massage sister.
She "was trained at Essex County Hospital, and has since
been pupil masseuse at the National Hospital, Queen
Square; and masseuse at the Royal Chest Hospital.
Harton Hospital, South Shields.?Miss Margaret Ellen
Keobano has been appointed sister. She was trained at
Stoke-on-Trent Workhouse Infirmary, and has since been
staff nurse at Epsom War Hospital and ward sister at her
training-school. She has also done private maternity work.
Kent and Canterbury Hospital, Canterbury.?Miss V.
Blake has been appointed eye sister and Miss F. Roberts
sister of the medical and eye wards. Both were trained at
the Royal Devon and Exeter Hospital.
Queen Victoria Royal Infirmary, Preston.?Miss Ger-
trude Moggoon has been appointed assistant matron. She
was trained at Bradford Royal Infirmary, where she was
later ward sister and night superintendent. She has also
been surgical ward sister at the West London Hospital,
Hammersmith, and night superintendent and assistant
matron at the Royal Hospital for Sick Children, Aberdeen.
West Bromwich District Hospital.?Miss Annie Charles-
worth has been appointed matron. She was trained at
Huddersfield Royal Infirmary, where she was later night
sister. She lias been sister at the General Hospital, Yar-
mouth, and assistant matron at Preston Royal Infirmary.
She was on active service in France and Italy under the
T.F.N.S., and was twice mentioned in dispatches. She
returned to Preston in 1919.
North Staffordshire Infirmary, Stoke-on-Trent.?Miss
Ellen Nix$n has been appointed sister. She was trained
at the North Staffordshire Infirmary, and has since done
private nursing. She was later sister at St. Saviour's Hos-
pital, Osnaburgh Street, N.W.
The Medical Department of the Board of
Education.
Thk Medical Branch of the Board of Education has been
transferred from Cleveland House, St. James's Square.
S.W. 1, to Bridgewater House, Cleveland Square, S.W. 1.
(Telephone No. : Gerrard 3410.)

				

